# From Diagnosis to Treatment: Exploring the Latest Management Trends in Cervical Intraepithelial Neoplasia

**DOI:** 10.7759/cureus.50291

**Published:** 2023-12-10

**Authors:** Saloni Gupta, Nikhilesh Nagtode, Vaibhav Chandra, Kavita Gomase

**Affiliations:** 1 Community Medicine, Jawaharlal Nehru Medical College, Datta Meghe Institute of Higher Education and Research, Wardha, IND; 2 Obstetrics and Gynecology, Smt. Radhikabai Meghe Memorial College of Nursing, Datta Meghe Institute of Higher Education and Research, Wardha, IND

**Keywords:** treatment decision-making, precision medicine, hpv vaccination, cin management, early detection, cervical intraepithelial neoplasia (cin)

## Abstract

Cervical intraepithelial neoplasia (CIN) stands as a precancerous condition with the potential to progress to invasive cervical cancer. This comprehensive review explores the intricacies of CIN management, beginning with its definition, classification, and etiology. It emphasizes the significance of early detection and outlines the latest trends in diagnosis, including Pap smears, human papillomavirus (HPV) testing, and colposcopy. Grading and staging, pivotal in treatment selection, are elucidated. Current management approaches, encompassing watchful waiting, surgical interventions, emerging minimally invasive techniques, and immunotherapy, are detailed. The factors influencing treatment decisions, informed consent, and patient education are discussed. Potential complications following treatment, the importance of long-term follow-up, and the role of HPV vaccination in prevention are underscored. Finally, the review looks to the future, discussing advances in detection, novel treatments, and the promise of precision medicine. In conclusion, early detection and management remain the cornerstone of CIN care, offering hope for a future where cervical cancer is a preventable and treatable condition.

## Introduction and background

Cervical intraepithelial neoplasia (CIN) is a precancerous condition of the cervix that has garnered significant attention in gynecology and oncology due to its potential to progress to invasive cervical cancer. This review article aims to comprehensively explore the latest management trends in CIN, shedding light on the evolving approaches from diagnosis to treatment [1,2].

The prevalence of CIN varies in different populations. A study conducted in Minia Maternity University Hospital in Egypt found that the prevalence of CIN was 3.3%, 0.84%, and 0.27% for CIN I, CIN II, and CIN III, respectively, and the prevalence of CIN II or higher was 1.11% [2]. Another study in China reported an overall crude high-risk human papillomavirus (HPV) prevalence of 17.7% [3]. In Tanzania, the prevalence of CIN is quite variable, ranging from 2.9% to 38% [3]. Regional differences in the prevalence of HPV types among Japanese women with CIN were also reported [4]. The prevalence of CIN varies in different populations and regions, and screening programs can help in the prevention of cervical cancer.

The significance of early detection and management of CIN cannot be overstated. Cervical cancer is a major global health concern, substantially impacting women's morbidity and mortality. However, the natural history of cervical cancer provides a unique opportunity for intervention. CIN lesions can be detected and treated before they progress to cancer, thereby preventing the development of a life-threatening malignancy. Implementing effective screening programs and timely interventions has significantly reduced cervical cancer incidence and mortality in many parts of the world [4].

This comprehensive review aims to critically examine the latest trends and advancements in managing CIN, a precancerous condition of the cervix. By providing a thorough overview of CIN, including its definition, classification, etiology, and risk factors, this review aims to lay the foundation for understanding the evolving approaches to its diagnosis and treatment. We will explore the current management strategies, ranging from conservative measures to surgical interventions and emerging minimally invasive techniques, while also discussing the role of immunotherapy in CIN treatment. Furthermore, we will delve into the complex treatment decision-making process, considering various patient-specific factors. Addressing potential complications and the importance of long-term follow-up and surveillance will be emphasized, along with the role of HPV vaccination as a preventive measure. Last, by examining the future directions and ongoing research in CIN management, this review aspires to contribute to the broader efforts to reduce the global burden of cervical cancer and enhance the quality of care for those affected by this condition.

## Review

Understanding CIN

Definition and Classification

CIN, or cervical dysplasia, is characterized by abnormal cellular changes within the epithelial lining of the cervix. It is typically classified into three grades: CIN 1, CIN 2, and CIN 3. CIN 1 represents mild dysplasia with relatively minor cellular abnormalities, while CIN 2 and CIN 3 indicate moderate to severe dysplasia with progressively higher degrees of cellular aberrations. Understanding this classification is essential for determining appropriate management strategies [1].

*Etiology* *and Risk Factors*

The etiology of CIN is predominantly attributed to high-risk HPV infections, specifically HPV-16 and HPV-18. HPV is a common family of viruses that infect the epithelial cells of the skin and mucous membranes, including those of the cervix. Among the vast array of HPV types, high-risk variants are particularly concerning due to their association with the development of cervical cancer and its precursors, CIN [5]. HPV is primarily transmitted through sexual contact, making sexual activity a key vector for infection. Early initiation of sexual activity and engagement with multiple sexual partners elevate the risk of encountering high-risk HPV types. This heightened risk can be attributed to increased opportunities for exposure to the virus [6].

Immunosuppression, whether due to medical conditions or medications, can compromise the body's ability to clear HPV infections effectively. As a result, individuals with weakened immune systems are at an elevated risk of persistent HPV infection and subsequent CIN development [7]. Cigarette smoking has also been identified as a significant risk factor for CIN. Smoking may impair the immune system's ability to combat HPV infections and accelerate the progression of cervical lesions [8].

Recognizing these risk factors is of paramount importance in the realm of CIN prevention and early detection. Public health initiatives and healthcare providers emphasize the significance of HPV vaccination as a preventive measure, particularly for adolescents and young adults. Additionally, awareness campaigns promote safe sexual practices, including the use of barrier methods like condoms, to reduce the transmission of HPV. Regular screenings and cervical cancer awareness programs target early detection, enabling timely intervention and preventing CIN progression to more advanced stages of invasive cancer [1].

Pathogenesis of CIN

HPV infection: The pathogenesis of CIN initiates by introducing high-risk HPV types into the cervical epithelium through sexual contact. HPV, a DNA virus, exhibits a particular predilection for mucosal epithelial cells, including those that line the cervix. Upon transmission, HPV establishes itself within these cells, setting the stage for a cascade of events that can culminate in CIN [9].

Viral entry and integration: Once introduced into the cervical epithelium, HPV targets susceptible cells through micro-abrasions or disruptions in the epithelial barrier, providing the virus access to the basal epithelial layer. Within this layer, HPV can integrate its genetic material into the host cell's DNA. This integration event can result in persistent infection, a hallmark of high-risk HPV infections and a significant contributor to CIN development [10].

E6 and E7 oncogenes: High-risk HPV types, such as HPV-16 and HPV-18, express viral oncoproteins known as E6 and E7, which play a central role in the pathogenesis of CIN. E6 and E7 act as molecular saboteurs, undermining the normal regulatory mechanisms of infected cervical cells. E6 targets the tumor suppressor protein p53, leading to its degradation. This hinders the cell's ability to arrest the cell cycle or undergo apoptosis in response to DNA damage, thereby permitting the survival of cells with genetic abnormalities. E7, on the other hand, interferes with the function of the retinoblastoma protein, promoting uncontrolled cell division and further contributing to cellular transformation [11].

Cellular transformation: The expression of E6 and E7 oncoproteins is pivotal in transforming cervical epithelial cells. These infected cells exhibit an increased proliferation rate, evading the normal regulatory mechanisms that control cell division. This uncontrolled growth is a hallmark of precancerous lesions, and without intervention, it can lead to the formation of CIN [12].

Genetic alterations: The continued presence of high-risk HPV and the expression of E6 and E7 oncoproteins can induce genetic alterations in infected cervical cells. These alterations may manifest as mutations in critical genes or chromosomal instability. These genetic changes can further disrupt the normal cellular regulatory pathways and contribute to the progression of cellular abnormalities [13].

Progression to CIN: Over time, the cumulative effects of HPV infection, oncoprotein expression, and genetic alterations can result in the development of CIN. CIN is categorized into different grades (CIN 1, CIN 2, and CIN 3) based on the extent and severity of cellular abnormalities observed within cervical tissue samples. This grading system is instrumental in guiding clinical decisions regarding the management and treatment of CIN [14].

Screening and Diagnosis

Pap smear (Papanicolaou test): The Pap smear, also known as the Papanicolaou test or Pap test, is a time-tested and widely employed screening method for the early detection of CIN and cervical cancer. The procedure involves collecting a sample of cervical cells during a pelvic examination. These cells are then examined under a microscope to detect cellular abnormalities, such as cell size, shape, or appearance [15]. The Pap smear has been instrumental in cervical cancer prevention by identifying precancerous changes in cervical cells. Depending on the results, healthcare providers can categorize findings into different groups, including "normal," "atypical squamous cells of undetermined significance (ASC-US)," "low-grade squamous intraepithelial lesion (LSIL)," and "high-grade squamous intraepithelial lesion (HSIL)." Abnormal results, particularly HSIL, often prompt further evaluation and management [16].

HPV testing: HPV testing involves the identification of high-risk HPV types in cervical samples. It is often used with the Pap smear to enhance CIN risk assessment. HPV testing is typically performed on the sample collected during the Pap smear [12]. This testing provides valuable information about a patient's risk for developing CIN, as persistent high-risk HPV infections are a major factor in CIN pathogenesis. When combined with Pap smear results, HPV testing can refine risk stratification and guide clinical decision-making. Individuals who test positive for high-risk HPV types, particularly HPV-16 and HPV-18, may undergo further evaluation, such as colposcopy [17].

Colposcopy and biopsy: When abnormalities are detected during Pap smears or HPV testing, colposcopy is often the next step in the diagnostic process. Colposcopy is a specialized procedure that involves using a magnifying instrument called a colposcope to examine the cervix more closely. It allows healthcare providers to visualize suspicious areas on the cervix's surface, such as abnormal blood vessels or lesions [18].

If suspicious lesions are identified during colposcopy, a biopsy may be performed. A small tissue sample is collected from the affected area for further examination during a cervical biopsy. The biopsy results can confirm the presence and grade of CIN, helping to guide treatment decisions [19]. The combination of these screening and diagnostic methods, along with a thorough clinical evaluation, enables healthcare providers to accurately identify CIN at different stages of development. Early detection is essential for initiating timely interventions, reducing the risk of CIN progression to invasive cervical cancer, and ensuring the best possible outcomes for affected individuals [20].

Grading and staging of CIN

CIN Grading System

CIN 1 (mild dysplasia): CIN 1 represents the presence of mild cellular abnormalities within the cervical epithelium. In this grade, the changes are typically confined to the lower one-third of the cervical epithelium. While these abnormalities are considered precancerous, they are relatively limited in extent and pose a lower risk of progressing to invasive cervical cancer. As a result, healthcare providers often employ a conservative approach, such as watchful waiting, to manage CIN 1, as many cases may spontaneously regress without intervention [21].

CIN 2 (moderate dysplasia): CIN 2 indicates moderate cellular abnormalities in the cervical epithelium. In this grade, the dysplastic changes extend deeper into the cervical tissue, covering the lower two-thirds of the epithelium. Although still categorized as precancerous, CIN 2 lesions are considered to have a higher degree of severity than CIN 1. These abnormalities are less likely to regress spontaneously and may progress to invasive cancer if left untreated. Therefore, intervention, such as surgical excision, is often recommended to remove or treat CIN 2 lesions and reduce the risk of progression [22].

CIN 3 (severe dysplasia/carcinoma in situ): CIN 3 represents the most severe form of cervical dysplasia and is often called carcinoma in situ. In this grade, cellular abnormalities are extensive, affecting the full thickness of the cervical epithelium. However, crucially, these abnormalities have not yet invaded the underlying connective tissue. CIN 3 is a high-risk condition that necessitates prompt and aggressive intervention to prevent the development of invasive cervical cancer. Treatment options may include surgical procedures such as excision or ablation to remove the abnormal tissue completely [23].

Differentiating Between CIN Grades

Accurate differentiation between CIN grades is crucial because it determines the appropriate course of action. The differentiation is typically based on histopathological examination of cervical tissue samples obtained through biopsies. Pathologists assess cellular features, including cell maturation, nuclear size, and mitotic activity, to distinguish between CIN grades [24].

Precise differentiation can be challenging, as there is subjectivity involved in histopathological interpretation. However, efforts are made to ensure interobserver agreement through standardized criteria and pathologist training. Additionally, emerging technologies, such as digital pathology and molecular markers, may aid in improving the accuracy of CIN grading [25].

Importance of Accurate Staging

Treatment selection:* *The cornerstone of accurate staging in CIN lies in its profound impact on treatment selection. CIN grading directly corresponds to the severity of cellular abnormalities within the cervical epithelium, which dictates the most appropriate therapeutic approach [26]. A conservative stance may be advisable in cases of milder CIN (CIN 1), where cellular abnormalities are confined to the lower one-third of the cervical epithelium and possess a lower risk of progression. This can encompass strategies like watchful waiting and diligent monitoring, recognizing that many CIN 1 lesions naturally regress without intervention [27].

In contrast, moderate CIN (CIN 2), characterized by abnormalities extending into the lower two-thirds of the cervical epithelium, often necessitates more assertive interventions. Healthcare providers frequently recommend surgical procedures such as excision or ablation to remove or treat CIN 2 lesions, significantly diminishing the risk of progression to invasive cervical cancer [28]. Severe CIN (CIN 3), the highest grade of cervical dysplasia, demands immediate and decisive treatment. While it has not yet infiltrated the underlying connective tissue, CIN 3 carries a substantial risk of evolving into invasive cancer if left unaddressed. Consequently, treatment approaches are typically aggressive and may entail completely removing the affected tissue [29].

Patient counseling:* *The precision of staging, derived from accurate grading, is instrumental in fostering effective patient counseling. When healthcare providers can convey to patients a clear understanding of the nature and severity of their condition, it lays the foundation for informed decision-making [30].

Empowered by knowledge about their CIN grade, patients can grasp the urgency and necessity of potential interventions. They become active participants in decision-making, weighing the risks and benefits of various treatment options. Accurate counseling enhances patient autonomy and engenders a sense of agency and ownership over their healthcare journey [31].

Prognostication:* *Staging further extends its critical role in prognostication, offering insights into the future trajectory of the disease. The severity of CIN grade is a harbinger of the likelihood of disease progression. CIN 3, with its extensive cellular abnormalities spanning the full thickness of the cervical epithelium, carries a significantly higher risk of advancing to invasive cervical cancer if allowed to persist untreated [32]. This stark reality underscores the imperative for timely intervention in CIN 3 cases. Healthcare providers must act swiftly to halt the progression of CIN, preventing the development of a potentially life-threatening malignancy. Prognostication guides the urgency of intervention and reinforces the pivotal role of accurate staging in CIN management [33].

Current management approaches

Watchful Waiting and Monitoring

Watchful waiting: Watchful waiting is a strategy that finds its place when dealing with CIN 1. This approach acknowledges that CIN 1 lesions have a relatively higher likelihood of regressing spontaneously without immediate intervention, especially in younger individuals. Therefore, healthcare providers may recommend watchful waiting rather than opting for invasive treatments upfront. The patient's condition is closely observed during this period without immediate medical interventions [34]. The rationale behind watchful waiting in CIN 1 cases lies in the recognition that not all mild dysplastic changes progress to advanced stages. A significant proportion of CIN 1 lesions may regress over time, returning the cervical tissue to its normal state. By avoiding unnecessary interventions, patients can potentially avoid treatments' associated risks and side effects [34].

Monitoring: While watchful waiting implies patience, it does not signify neglect. Rigorous monitoring is an integral aspect of this strategy. Patients undergoing watchful waiting are typically advised to participate in a structured surveillance program. This monitoring involves regular follow-up appointments and specific tests, such as Pap smears or HPV testing [35]. Close surveillance aims to track the status of CIN lesions over time. If, during monitoring, the lesions persist or demonstrate signs of progression, further intervention may be warranted. In such cases, the transition to more active treatment approaches, such as surgical excision, may be initiated promptly to address the evolving condition effectively [35]. Monitoring not only allows healthcare providers to detect any concerning changes in a timely manner but also assures patients that their condition is being carefully evaluated. This proactive approach ensures that if intervention becomes necessary, it can be undertaken at an optimal juncture, minimizing the potential for disease advancement [35].

Surgical Interventions

Loop electrosurgical excision procedure: Loop electrosurgical excision procedure (LEEP) is a minimally invasive surgical technique designed to excise abnormal cervical tissue using a thin, electrified wire loop. This procedure is widely employed for treating CIN, particularly when the lesion extends into the cervical canal. The loop, guided by a colposcope, precisely removes the affected tissue, allowing for diagnostic and therapeutic purposes. LEEP boasts several advantages, including its outpatient setting, rapid recovery, and minimal discomfort. It effectively targets and eliminates dysplastic tissue, preventing its progression while preserving the structural integrity of the cervix. LEEP's versatility and efficacy have made it a mainstay in the treatment armamentarium for CIN [36].

Cold knife conization: Cold knife conization represents a more extensive surgical procedure for managing CIN. It involves the surgical removal of a cone-shaped section of the cervix using a scalpel or laser. This technique is typically reserved for more severe cases of CIN, particularly when suspected of invasive disease. Cold knife conization offers the advantage of obtaining larger tissue specimens for histopathological examination, allowing for precise diagnosis and evaluation of disease extent. However, it is associated with a longer recovery time and a greater risk of adverse effects than less invasive procedures. Its use is carefully considered based on the clinical presentation and extent of the cervical lesion [37].

Laser therapy: Laser therapy harnesses the power of a high-energy beam of light to vaporize and remove abnormal cervical tissue. This precise and effective treatment option is often chosen for certain CIN 2 and CIN 3 cases. Laser therapy offers the advantage of minimal blood loss, reduced scarring, and quicker recovery times. It is particularly well-suited for lesions that are not amenable to excision by other means, such as those located near the cervical os or within the endocervical canal. The ability to precisely target and ablate abnormal tissue makes laser therapy a valuable tool in the therapeutic arsenal for CIN [38].

Emerging minimally invasive techniques

Cryotherapy

Outpatient procedure: Cryotherapy is notably an outpatient procedure, a hallmark of accessibility and convenience. This characteristic spares patients the need for hospitalization or extended stays, aligning with the modern paradigm of healthcare that prioritizes minimizing the burden on patients and healthcare systems alike. Avoiding hospital admissions reduces healthcare costs, rendering CIN treatment more economically viable. Furthermore, outpatient cryotherapy enhances the accessibility of CIN treatment to a broader population, making it available to individuals who might otherwise face logistical or financial barriers to healthcare. This inclusivity is a testament to the egalitarian approach of cryotherapy in addressing CIN [39].

Minimal discomfort: A pivotal facet of cryotherapy is the minimal discomfort experienced by patients during the procedure. Local anesthesia is typically sufficient to manage any sensations of cold or mild cramping associated with the treatment. This aspect contributes significantly to a more comfortable treatment experience for patients. The reduced discomfort minimizes anxiety and apprehension that patients may associate with medical procedures, fostering a more positive and less stressful encounter with healthcare. It exemplifies the patient-centered ethos of cryotherapy, where the patient's physical and emotional well-being is central to the treatment process [40].

Quick recovery: One of the most remarkable advantages of cryotherapy is its swift recovery period. Most patients can typically resume their regular activities without significant delay after the procedure. This rapid return to normalcy is a testament to the minimally invasive nature of cryotherapy. It minimizes disruptions to patients' daily lives, allowing them to maintain their routines and responsibilities. This aspect enhances patient satisfaction and underscores the patient-centric philosophy of minimally invasive techniques. Patients can swiftly regain control over their lives, free from the protracted recovery periods that may accompany more invasive procedures [41].

Suitability: Cryotherapy's suitability for selected cases of CIN further highlights its versatility within the spectrum of CIN management. It is particularly well-suited for treating lesions of lesser severity, such as CIN 1 or early-stage CIN 2. In these instances, cryotherapy offers a conservative yet effective option for managing precancerous lesions. Importantly, it does so while preserving crucial aspects of cervical function and fertility in many cases. This adaptability ensures that cryotherapy can be tailored to the individual needs and circumstances of patients. It aligns with personalized medicine principles, where treatments are customized to maximize patient benefits while minimizing adverse impacts [42].

Thermal Ablation

Less invasiveness: One of the hallmark advantages of thermal ablation methods is their significantly reduced invasiveness when compared to traditional surgical approaches. These techniques involve heat's precise and targeted application to the affected tissue. This precision minimizes collateral damage to healthy surrounding tissue, a critical factor in preserving cervical function and minimizing potential side effects. The minimization of invasiveness enhances patient safety during the procedure and contributes to a more comfortable experience. Patients can undergo thermal ablation with greater confidence, knowing that the treatment aims to be minimally disruptive to their overall health and well-being [43].

Effective tissue removal: Thermal ablation techniques are highly effective in removing abnormal cervical tissue. The controlled application of heat serves to denature and destroy precancerous cells within the cervical epithelium. This precision ensures that the targeted tissue is effectively eliminated, reducing the risk of residual abnormalities or recurrences. The efficacy of thermal ablation techniques in removing abnormal tissue aligns with CIN management's overarching goal of preventing precancerous lesions' progression to invasive cervical cancer. This high level of effectiveness promotes successful treatment outcomes and instills patients' confidence regarding the therapeutic approach [44].

Reduced recovery time: Patients who opt for thermal ablation typically experience shorter recovery times than those undergoing traditional surgical interventions. The abbreviated recovery period is a substantial boon to patients' overall quality of life. It enables individuals to return to their usual activities sooner, minimizing disruptions to their daily routines and responsibilities. This aspect of thermal ablation aligns with the patient-centered ethos of modern healthcare, emphasizing the importance of restoring patients to their regular lives promptly and with minimal interruption. The reduced recovery time underscores the practicality and patient-friendliness of thermal ablation techniques, making them an attractive option for individuals seeking effective CIN treatment without lengthy recuperation periods [45].

Immunotherapy and CIN

Immunotherapy is a promising avenue in CIN management. It involves using immune-based treatments to target and eliminate abnormal cervical cells. One such immunotherapy approach is therapeutic vaccines designed to stimulate the immune system's response against HPV and CIN-related lesions. Clinical trials are ongoing to evaluate the efficacy of immunotherapy in CIN treatment, and results are awaited with great anticipation [46].

Treatment decision-making: factors influencing treatment selection

Age of the Patient

Younger individuals with CIN 1: For younger patients, especially those diagnosed with CIN 1, watchful waiting may emerge as a viable option. This approach is grounded in recognizing that CIN 1 lesions frequently exhibit a propensity for spontaneous regression, primarily due to the robust immune responses often found in younger individuals. The potential for the lesions to resolve independently without medical intervention is a key consideration for these patients. A conservative approach allows healthcare providers to closely monitor the lesions for any signs of regression without immediately resorting to more invasive treatments. This approach embodies the principle of avoiding overtreatment, reducing potential risks, and respecting the body's capacity for self-healing [47].

Older patients or those with CIN 3: In contrast, older patients or those diagnosed with higher-grade CIN, such as CIN 3, are often recommended to pursue a more aggressive treatment approach. As age advances, there may be a diminished likelihood of spontaneous regression for CIN lesions, and the risk of CIN 3 progressing to invasive cervical cancer becomes significantly higher. Therefore, aggressive interventions, such as surgical procedures or ablation techniques, are typically favored to curtail the potential for disease progression. This approach underscores the importance of timely and decisive intervention in mitigating the risks associated with more advanced CIN [48].

CIN Grade and Extent

CIN 1 management: CIN 1 lesions, characterized by mild cellular abnormalities typically confined to the lower one-third of the cervical epithelium, are often managed conservatively. Conservative management may involve watchful waiting or less invasive interventions, given the relatively low risk of progression associated with CIN 1. This approach aligns with the goal of avoiding overtreatment while respecting the potential for spontaneous regression seen in these cases [49].

CIN 2 and CIN 3 management: In contrast, CIN 2 and CIN 3 dysplasia, encompassing more extensive cellular abnormalities affecting larger areas of the cervical epithelium, typically warrants more aggressive interventions. These higher-grade CIN lesions present an elevated risk of progressing to invasive cervical cancer if left untreated. Consequently, surgical procedures or ablation techniques are frequently recommended to ensure the complete removal of the abnormal tissue, thus reducing the risk of malignant transformation. The choice of intervention is tailored to the specific characteristics of the lesion to optimize treatment outcomes [23].

Fertility Considerations

Less invasive options for fertility preservation: Less invasive treatments, such as LEEP, are often favored when fertility preservation is a primary concern. LEEP selectively removes abnormal tissue while minimizing the impact on cervical integrity. This approach reduces the risk of cervical insufficiency, which can lead to pregnancy complications, including preterm birth. Patients can effectively address their CIN by opting for less invasive interventions while safeguarding their fertility aspirations [50].

Considerations for more extensive procedures: In contrast, more extensive procedures like cold knife conization or laser therapy may be chosen when fertility preservation is not the primary focus or when higher-grade CIN (e.g., CIN 3) necessitates a more aggressive treatment approach. While effective at removing abnormal tissue, these procedures can have a greater impact on cervical integrity and may increase the risk of pregnancy complications. In such cases, the potential consequences for fertility are weighed against the imperative of treating the precancerous condition [51].

Informed Consent and Patient Education

Explaining treatment options: Healthcare providers are responsible for elucidating the available treatment options in a clear, concise, and comprehensible manner. This explanation should encompass the procedural details, potential side effects, and expected recovery times associated with each treatment option. By providing a comprehensive overview of these facets, healthcare providers empower patients to make informed choices about their care [52].

Risks and benefits: Patients must be fully informed about each treatment modality's potential risks and benefits. This includes candid discussions about the risk of bleeding, infection, cervical stenosis, and, particularly in the case of surgical interventions, the potential impacts on future fertility and pregnancy. By delineating the advantages and disadvantages of each approach, healthcare providers enable patients to weigh the potential outcomes and make decisions that align with their health goals and concerns [53].

Alternatives: In line with the principle of informed consent, patients should be apprised of any viable alternatives to the proposed treatment. In some cases, watchful waiting may be an appropriate and evidence-based choice. By presenting alternatives, healthcare providers allow patients to consider various paths to managing their condition, fostering a sense of agency in their healthcare journey [54].

Patient preferences: Respecting and accommodating patient preferences and values is paramount in decision-making. Patients may have unique priorities and concerns, such as fertility preservation or optimizing the likelihood of complete CIN removal. Healthcare providers should actively engage with patients to understand their preferences and collaborate in tailoring a treatment plan that aligns with these preferences. This collaborative approach ensures that healthcare decisions align with the individual's values and priorities [55].

Supportive care: The informed consent process and patient education extend beyond the initial treatment decision. Patients should receive comprehensive information about post-treatment care and follow-up protocols. Emphasis should be placed on the importance of regular screenings to monitor for recurrence and ensure the early detection of any issues. This ongoing education equips patients with the knowledge and tools needed to participate in their long-term health management actively [56].

Complications and follow-up

Potential Complications of CIN Treatment

Bleeding: One of the most common complications following CIN treatment is vaginal bleeding. This can manifest as mild spotting or, in some cases, more significant bleeding, particularly after surgical interventions such as LEEP or conization. The severity of bleeding can vary among individuals. Healthcare providers must emphasize the importance of post-treatment monitoring and guide what to expect. Patients should be informed when to seek immediate medical attention if bleeding exceeds normal post-treatment expectations. Additionally, patients should be educated about the importance of avoiding activities that could exacerbate bleeding, such as sexual intercourse or tampons during the post-treatment recovery period [57].

Infection: Although relatively rare, infection can occur after CIN treatment. Patients should be educated on the signs of infection, including increased pain, fever, or abnormal discharge, and advised to seek prompt medical attention if these symptoms arise. Preventive measures, such as post-treatment antibiotics, may be prescribed in certain cases to reduce the risk of infection. Patient education should underscore the significance of adhering to prescribed post-treatment care instructions to minimize the likelihood of infections [57].

Cervical stenosis: Surgical procedures for CIN treatment, particularly cold knife conization, carry a risk of cervical stenosis. This condition involves the narrowing or partial closure of the cervical canal. Cervical stenosis can lead to menstrual abnormalities, such as heavier or more painful periods, and may also pose challenges for fertility. Patients should be informed about this potential complication, and the implications for their menstrual health and reproductive goals should be discussed as part of the decision-making process. Alternative treatment options that minimize the risk of cervical stenosis may be considered, especially for individuals who prioritize fertility preservation [37].

Preterm birth risk: In cases where a significant portion of the cervix is removed during CIN treatment, there may be an increased risk of preterm birth in subsequent pregnancies. This risk arises from the potential weakening of the cervix due to the removal of cervical tissue. It is essential to consider fertility implications when selecting treatment options, especially for patients who plan to have children in the future. Patients should receive comprehensive information about the potential risks and benefits of different treatments, considering their fertility aspirations [58].

Long-Term Follow-Up and Surveillance

Monitoring for recurrence: Regular follow-up appointments and screenings constitute the cornerstone of long-term CIN management. This includes periodic Pap smears and HPV testing, essential for monitoring any signs of CIN recurrence. These screenings aim to detect cellular abnormalities or persistent HPV infections that may signal the return of precancerous lesions. Early detection through regular monitoring is imperative, as it enables timely intervention and minimizes the risk of disease progression [59].

Addressing complications: Long-term follow-up also encompasses the management of potential complications that may arise post-treatment. Patients undergoing CIN treatment, particularly surgical procedures, may experience complications such as cervical stenosis or persistent bleeding. Timely recognition and intervention are crucial to address these issues effectively. Healthcare providers should actively assess and manage complications, ensuring the patient's comfort and well-being [48].

Cervical cancer prevention: The primary objective of long-term follow-up and surveillance is to prevent the progression of CIN to invasive cervical cancer. Continuous monitoring allows healthcare providers to track the patient's cervical health over time and intervene promptly if abnormalities or recurrences are detected. Identifying and addressing precancerous changes at an early stage reduces the risk of invasive cancer development. This preventive aspect of long-term surveillance underscores its vital role in preserving the patient's health [60].

Counseling and support: Long-term follow-up provides an invaluable opportunity for healthcare providers to offer counseling and support to patients. Patients may be concerned about their cervical health, treatment outcomes, or potential future pregnancies. Addressing these concerns and providing guidance on maintaining cervical health is essential. Additionally, healthcare providers can offer emotional support, addressing any anxiety or apprehension patients may experience during the follow-up process. Patient education and support contribute to a more holistic and patient-centered approach to CIN management [61].

Role of HPV Vaccination

Primary prevention: HPV vaccines are most effective when administered before individuals are exposed to HPV. Therefore, a primary prevention strategy entails vaccinating adolescents and young adults. Targeting this age group aims to significantly reduce the incidence of HPV infections, which are the underlying cause of CIN and cervical cancer. The vaccines create immunity against specific HPV types, preventing the initial infection and reducing the risk of developing precancerous cervical lesions [62].

Vaccine types: Several HPV vaccines offer protection against different HPV types, including the high-risk types most closely linked to CIN and cervical cancer. Healthcare providers play a crucial role in educating patients about the benefits of HPV vaccination and recommending the most appropriate vaccine based on individual factors such as age and sex. Patients should be well-informed about completing the recommended vaccine series to achieve optimal protection [63].

Vaccination across the lifespan: HPV vaccination is not confined to a specific age group; its impact extends across the lifespan. While the primary focus is on vaccinating adolescents and young adults, there are important considerations for individuals who were not vaccinated during their adolescent years. Catch-up vaccination is recommended for those who missed the opportunity for vaccination in adolescence. Additionally, some countries have expanded HPV vaccination programs to include older age groups to maximize its public health impact. These efforts aim to extend the benefits of HPV vaccination to a broader population, reducing the overall burden of CIN and cervical cancer [64].

Future directions and research

Advances in CIN Detection and Diagnosis

Liquid biopsies: Liquid biopsy techniques are at the forefront of research for detecting CIN and monitoring disease progression. These non-invasive methods involve the analysis of various biomolecules, such as circulating tumor DNA and microRNA, in bodily fluids like blood or cervical secretions. Liquid biopsies offer the potential for earlier and more convenient detection of CIN, reducing the need for invasive procedures like colposcopy and biopsy. Additionally, they may provide insights into the molecular characteristics of CIN, enhancing our understanding of the disease [65].

Advanced imaging technologies: Emerging imaging technologies are revolutionizing the visualization of cervical abnormalities, improving diagnostic accuracy, and minimizing the need for invasive biopsies. Optical coherence tomography (OCT) and high-resolution microendoscopy are among the cutting-edge techniques being explored. OCT offers high-resolution, real-time imaging of cervical tissue, allowing healthcare providers to visualize cellular and structural changes. On the other hand, high-resolution microendoscopy provides detailed images of the cervix's surface, aiding in identifying precancerous lesions. These advanced imaging methods can potentially streamline the diagnostic process and enhance patient comfort [66].

Biomarker discovery: Ongoing research endeavors aim to identify novel CIN-associated biomarkers. Biomarkers are specific molecules or genetic markers that indicate disease presence, progression, or response to treatment. Discovering and validating CIN-specific biomarkers can lead to more specific and reliable diagnostic tests. Furthermore, biomarkers may play a role in predicting an individual's response to various treatment modalities, enabling personalized therapeutic approaches. Developing biomarker-based assays could significantly improve the accuracy and efficiency of CIN diagnosis and management [67].

Novel Treatment Modalities

Topical therapies: Researchers are actively investigating topical therapies as potential treatments for CIN. These therapies encompass a range of agents, including immune modulators and photodynamic therapy. Topical treatments aim to precisely target and eliminate abnormal cervical tissue while sparing healthy cells. Immune modulators, such as imiquimod, stimulate the body's immune response to target and clear precancerous lesions. Photodynamic therapy involves using photosensitizing agents and light to destroy abnormal cells selectively. These approaches offer the advantage of being minimally invasive and may reduce the need for surgical interventions, preserving cervical function and fertility in many cases [68].

Targeted therapies: Targeted therapies represent a promising avenue to combat CIN. These treatments are designed to disrupt specific molecular pathways involved in the development of CIN. By precisely targeting the underlying mechanisms driving disease progression, targeted therapies offer the potential for increased treatment efficacy and reduced side effects compared to traditional approaches. Precision medicine approaches may help identify patients most likely to benefit from these treatments based on their unique genetic and molecular profiles [69].

Gene editing technologies: Emerging gene editing technologies, such as CRISPR-Cas9, hold significant promise in CIN treatment. While still in the early stages of development, these techniques offer the potential to target and correct genetic abnormalities in cervical cells precisely. Gene editing technologies aim to restore normal cellular function and halt the progression of CIN. The specificity and precision of these approaches are particularly appealing, as they can potentially spare healthy tissue and minimize adverse effects associated with more invasive treatments [70].

Precision Medicine and CIN

Personalized treatment: Precision medicine involves tailoring CIN treatment to the unique characteristics of each patient. Genetic profiling and molecular analysis are essential components of this approach. By understanding the genetic and molecular underpinnings of CIN in an individual, healthcare providers can identify the most effective treatment options while minimizing potential side effects. Personalized treatment plans ensure that patients receive interventions optimized for their specific CIN profile, improving treatment efficacy and patient satisfaction [71].

Immunotherapy advancements: Immunotherapy has gained prominence in the field of CIN treatment, and precision medicine is at the forefront of these advancements. Researchers are continuously exploring the development of therapeutic vaccines and immune checkpoint inhibitors specifically designed to target CIN-related lesions. These immunotherapeutic approaches harness the body's immune system to target and eliminate precancerous cells, offering a promising alternative to traditional treatments. Precision medicine enables the identification of patients who are most likely to benefit from these innovative immunotherapies based on their individual immune profiles [72].

Risk stratification: Precision medicine also plays a pivotal role in risk stratification for CIN. By analyzing an individual's genetic and molecular characteristics, healthcare providers can identify those at the highest risk of CIN progression. This information informs more proactive intervention and surveillance efforts for high-risk individuals, ensuring that resources are allocated efficiently to those who need them the most. Risk stratification is instrumental in optimizing the allocation of healthcare resources and improving patient outcomes [73].

Patient-centered care: A fundamental principle of precision medicine is its patient-centered approach. It empowers individuals with comprehensive information about their disease, including genetic and molecular insights, and provides them with a central role in shared decision-making. Patients become active participants in their care, collaborating with healthcare providers to make informed choices about treatment options, considering their unique circumstances, values, and preferences. This patient-centered approach enhances the overall patient experience and fosters a sense of agency and ownership in managing their cervical health [74].

## Conclusions

In conclusion, managing CIN represents a critical frontline defense in the fight against cervical cancer. This review has underscored the importance of early detection and proactive intervention, highlighting the potential for CIN to progress to a life-threatening malignancy if left untreated. We have explored the complexities of CIN, from its classification and diagnosis to the various treatment modalities available today. The evolving landscape of CIN management, marked by advances in detection, novel treatment options, and the promise of precision medicine, offers hope for more effective, personalized, and less invasive approaches. Furthermore, this review has emphasized the significance of patient-centered care, informed decision-making, and the role of HPV vaccination in the broader context of CIN and cervical cancer prevention. As we look ahead, optimism fills the horizon with the prospect of reducing the global burden of cervical cancer and improving the lives of countless individuals through continued research, innovation, and patient-focused care.
